# The clinical characteristics of COVID-19 omicron variant infection in pregnant women and their neonates

**DOI:** 10.3389/fmed.2023.1229794

**Published:** 2023-09-14

**Authors:** Li-Li Liu, Lv-Hua Lin, Fen Lin, Yi-Kang Yang, Chun-Fan Lin, Lin Zhang, Yu-Chan Huang, Yu-Wei Liao, Yan-Qing Zeng, Guang-Kuan Zeng, Yan-Bin Cao, Rui-Zhen Zhu, Li-Ye Yang

**Affiliations:** ^1^Precision Medical Lab Center, People’s Hospital of Yangjiang Affiliated to Guangdong Medical University, Yangjiang, Guangdong, China; ^2^Department of Obstetrics, People’s Hospital of Yangjiang Affiliated to Guangdong Medical University, Yangjiang, Guangdong, China; ^3^Precision Medical Lab Center, Chaozhou Central Hospital, Chaozhou, Guangdong, China; ^4^Institute of Medicine and Nursing, Hubei University of Medicine, Shiyan, Hubei, China; ^5^Department of Neonatology, People’s Hospital of Yangjiang Affiliated to Guangdong Medical University, Yangjiang, Guangdong, China; ^6^Key Laboratory of Respiratory Disease of Yangjiang, People's Hospital of Yangjiang Affiliated to Guangdong Medical University, Yangjiang, Guangdong, China; ^7^Yangjiang Branch of Biochip Beijing National Engineering Research Center, People's Hospital of Yangjiang Affiliated to Guangdong Medical University, Yangjiang, Guangdong, China

**Keywords:** COVID-19, omicron variant, pregnant women, neonate, China

## Abstract

**Objective:**

The objective of this study was to analyze the clinical characteristics of pregnant women infected with the COVID-19 omicron variant and their neonates during the outbreak in Guangdong province, China.

**Methods:**

The clinical data of pregnant women infected with the COVID-19 omicron variant and their neonates were retrospectively collected from two hospitals in Guangdong province. Information recorded included age of mother, date of birth, sex, weight at birth, mode of delivery, gestational age, feeding mode, Apgar score, signs, medical records, underlying comorbidities and laboratory results. The presence of SARS-CoV-2 viral RNA was tested using an real-time PCR assay.

**Results:**

Seventy-nine pregnant women infected with COVID-19 omicron variant and their 68 neonates were included in this study. The vast majority (86.1%) of pregnant women was in their third trimester of pregnancy, and only 11 cases (15%) were in the first or second trimester. Of 79 pregnant women, 39 cases were asymptomatic at the time of infection, and 40 mothers presented with mild manifestations of COVID-19. The most common symptoms were fever (92.5%, 37/40) and cough (57.5%, 21/40). All of pregnant women did not receive chest computed tomography (CT) scan or X-ray. No pregnant woman developed severe pneumonia. A total of 68 neonates (3 set of twins) from 65 mothers with COVID-19 were reviewed. Among women who delivered, 34 cases underwent cesarean section, 31 cases underwent vaginal delivery. According to the timing of birth, there were 10 (14.7%) preterm neonates. Two babies were born dead (intrauterine fetal death after 22 weeks of gestation). Of the live babies born (66 cases) from mothers with COVID-19, 9 newborns were lower weight, and one preterm case was born with respiratory distress and intubated, he recovered and developed normally. SARS-CoV-2 nucleic acid testing was conducted on 41 neonates daily after birth, with only one neonate testing positive for SARS-CoV-2 infection on the third day after birth. The infected neonate exhibited typical fever and acute respiratory tract syndrome but ultimately had a good prognosis, recovering after 5 days of treatment.

**Conclusion:**

Although preliminary data suggests the risk of severe maternal and fetal complications from Omicron variant infection during pregnancy is lower than previous variants and Delta variant. Our study, which was conducted on a limited population sample, indicates that there is a possibility of severe complications, such as stillbirth, occurring in some fetal cases. These findings emphasize the need for continued attention from obstetricians.

## Background

COVID-19 has been a global health concern since December 2019. In November 2021, a new variant of COVID-19, named SARS-CoV-2 omicron or B.1.1.529, was officially reported to the World Health Organization (WHO) by South Africa. This variant spreads significantly faster than previous variants, leading to a surge in cases in many countries. It has become the most prevalent SARS-CoV-2 variant worldwide (1).

Multiple variants of SARS-CoV-2 omicron have emerged and continue to evolve. Despite being the most complexly mutated variant thus far, its symptoms are generally mild. The SARS-CoV-2 omicron variant has shown a significantly lower number of severe cases leading to hospitalization or death compared to previous variants. This could be partly due to global vaccination efforts and natural immunity (2, 3). Although preliminary data suggests the risk of severe maternal and fetal complications from Omicron variant infection during pregnancy is lower than previous variants and Delta variant, given the fast transmission speed and wide affected population, its clinical implications still warrant attention from obstetricians (4).

There was no report regarding the clinical characteristics, pregnancy outcomes, and potential risks of vertical transmission of SARS-CoV-2 omicron variant infection in China. Given the unique characteristics of transmission and virulence observed in the omicron variant, along with its severity and mortality rates, this study aims to investigate the clinical characteristics, pregnancy outcomes, and potential risks of vertical transmission of SARS-CoV-2 infection during pregnancy.

## Methods

### Study population

From December 12, 2022 to January 14, 2023, pregnant women and their neonates admitted in People’s Hospital of Yangjiang (Tertiary Hospital, the biggest hospital in Yangjiang area) and Chaozhou Central Hospital (Tertiary Hospital in Chaozhou) were retrospectively reviewed. The diagnostic criteria for pregnant women with COVID-19 were: (1) pregnant; (2) positive for SARS-CoV-2 nucleic acid in nasal swab and (or) oropharynx swab. The presence of SARS-CoV-2 viral RNA was tested using an in-house Taqman rt-real-time PCR assay targeting N and ORF1ab genes. The date of disease onset was defined as the day a symptom was noticed. Demographic and clinical record, and laboratory results were reviewed and collected by the ordering obstetrician and pediatricians from electronic medical records. Information recorded included age of mother, date of birth, sex, weight at birth, mode of delivery, gestational age, feeding mode, Apgar score, signs, medical records and underlying comorbidities. Laboratory tests including blood routine, renal and hepatic function test were also reviewed. Chest imaging findings and outcomes were also collected.

COVID-19 diagnosis and classification criteria were based on “New coronavirus pneumonia diagnosis and treatment protocol (trial version 10),” which was issued by the national health commission of P. R. China (5).

This study was approved by the Ethics Committee of People’s Hospital of Yangjiang (20230003) and Chaozhou Central Hospital. As the patient’s data were analyzed anonymously and this was a retrospective study, a waiver of written consent was approved by the Ethics Committee of the two hospitals.

### Statistical analysis

All statistical analyses were performed using SPSS 20.0 software. Categorical variables were described as frequency and percentages, and continuous variables were described as mean ± SE. Differences in continuous variables were analyzed with independent group t-tests when the data were normally distributed. Differences in categorical variables within the groups were compared by Chi-square test or Fisher’s exact test as appropriate, and statistical significance was set as *p* < 0.05.

## Results

### Demographic and clinical characteristics of pregnant women with COVID-19

A total of 79 pregnant women with COVID-19 infection were included in the study. 73 cases were confirmed with COVID-19 infection on the admission day, 6 cases were infected during hospitalization. The median age of the study population was 30 years, and 7 (8.9%) women were aged ≥40 years (Table 1). There was significant difference for the ages between asymptomatic and symptomatic group (mean 28 years vs. mean 32 years, *P* < 0.001, Student’s *t*-test), symptomatic women showed higher mean age compared to asymptomatic women (Table 1). There was no significant difference for parity, trimester of SARS-CoV-2 infection, comorbidities, and vaccination status between asymptomatic and symptomatic group (Table 1).

**Table 1:** The characteristics of pregnant women with COVID-19 omicron variant.

Characteristics	Total pregnant women (*N* = 79)	Maternal disease severity asymptomatic (*N* = 39)	Maternal disease severity symptomatic (*N* = 40)	*p-*value
Age, year	Median 30 (Range: 18–42) (IQR: 27.33)	Median 28 (Range: 18–41) (IQR: 25.30)	Median 32 (Range: 18–42) (IQR: 28.36)	<0.001
Age ≥ 40 year	7	2	5	0.432
Parity				0.550
0	25	14	11	
1	23	12	11	
2	21	9	12	
≥3	10	4	6	
Trimester of SARS-CoV-2 infection				0.031
First trimester (<14 week)	5	2	3	
Second trimester (14–27 week)	6	0	6	
Third trimester (≥28 week)	68	37	31	
Comorbidities				0.515
Gestational hypertension	2	0	2	
Gestational diabetes	10	3	7	
Mild thalassemia	9	4	5	
Gestational hyperthyroidism	1	0	1	
Gestational hypothyroidism	1	1	0	
Intrahepatic cholestasis	4	2	2	
Double uterus and double vagina	1	1	0	
G6PD deficiency	4	1	3	
Idiopathic thrombocytopenic purpura	1	1	0	
Oviducal pregnancy	1	0	1	
Anemia	37	18	19	
Hysteromyoma	2	2	0	
IVF-ET	3	1	2	>0.999
Vaccination status				0.076
No	7	6	1	
One dose	1	1	0	
Two doses	29	11	18	
Three doses	42	21	21	

Ages are presented as median, range and interquartile range. IVF-ET, *in vitro* fertilization and embryo transplantation.

Among 79 pregnant women, 25 (31.6%) cases were nulliparous. The vast majority (86.1%, 68/79) of pregnant patients were in their third trimester of pregnancy, and only 11 cases were in the first (6.3%, 5/79) or second trimester (7.6%, 6/79). A total of 46 (58.2%) pregnant women had one or more underlying medical conditions, 37 cases had anemia with hemoglobin below 110 g/L, while 10 cases had gestational diabetes, nine cases had mild thalassemia, four cases had intrahepatic cholestasis, and 1 case had a double uterus and double vagina. Additionally, three cases underwent *in vitro* fertilization and embryo transplantation, as shown in Table 1.

Of 79 pregnant women, 39 cases (49.4%) were asymptomatic for COVID-19 at the time of infection. Forty cases (50.6%) presented with symptoms of COVID-19. The most common symptoms were fever (92.5%, 37/40), peak temperature was ≥39°C in 10 cases, and the other symptoms were cough (57.5%, 21/40), fatigue (32.5%, 13/40), dizziness and headache (20.0%, 8/40), nasal congestion (22.5%, 9/40), sore throat (20%, 8/40), poor appetite (15%, 6/40), runny nose (20%, 8/40), and vomiting (12.5%, 5/40).

Seventy two women were vaccinated for at least one dose, they were vaccinated from January 2021 to April 2022, and there was no difference statistically between the symptomatic group and the asymptomatic group for vaccination status (*p* = 0.076) (Table 1).

### Chest imaging and laboratory findings

No case had chest computed tomography (CT) scan or X-ray performed due to the mild presentation of COVID-19. As for the laboratory tests, the presence of SARS-CoV-2 viral RNA was confirmed in all 79 pregnant cases at the time of admission or during hospitalization. Of the 79 cases who had complete peripheral blood cell count tested, 17 (21.5%) had white blood cell (WBC) count >9.5 × 10^9^/L, the WBC count ranged from 4.08 × 10^9^/L to 14.61 × 10^9^/L. Increased neutrophils were observed in 32 cases (40.5%), lymphopenia occurred in 39 cases (49.4%) and thrombopenia was observed in 3 cases (3.8%). 46.8% (37/79) had anemia (Hemoglobin lower than 110 g/L). Of the 23 cases who had peripheral blood C-reactive protein (CRP) tested, 8 (34.8%) had CRP < 6 mg/L, 15 (65.2%) had CRP > 6 mg/L (range: 7.35–36.5 mg/L). Hepatic involvement included elevated ALT (11.4%, 9/79) and elevated AST (13.9%, 11/79). A mother with COVID-19 symptom delivered two babies, one was stillbirth (intrauterine fetal death), the other was born with respiratory distress, had elevated ALT (297.1 U/L) and elevated AST (149.9 U/L). Other laboratory abnormalities included hypoalbuminemia (96.2%, 76/79). Comparison of laboratory parameters was conducted between the symptomatic and asymptomatic groups, and it was found that only the lymphocyte count showed significant difference statistically, and there was no significant difference for other parameters (Table 2).

**Table 2:** Laboratory findings according to the severity classification in pregnant women with COVID-19.

Laboratory findings	Symptomatic (*n* = 40) n or Mean ± SD	Asymptomatic (*N* = 39) n or Mean ± SD	*p-*value^#^
WBC	8.18 ± 3.03	8.13 ± 2.69	0.938
>9.5	8	9	
Neutrophil	6.45 ± 2.69	6.08 ± 2.56	0.54
Lymphocyte	0.93 ± 0.66	1.35 ± 0.61	0.005
RBC	4.01 ± 0.68	4.06 ± 0.66	0.715
HGB	110.38 ± 19.05	116 ± 23.68	0.248
<110	20	17	
<90	3	1	
Platelet	225.3 ± 71.29	209.54 ± 66.79	0.314
ALT	35.58 ± 52.89	18.98 ± 28.24	0.099
>40	8	1	
>80	4	1	
AST	35.01 ± 32.68	23.87 ± 27.74	0.119
>35	10	1	
>70	4	1	
ALB	34.61 ± 3.13	34.37 ± 2.92	0.726
<40	37	35	
GLB	31.06 ± 3.76	30.94 ± 3.41	0.888
>40	1	1	
Urea	2.66 ± 0.81	3.18 ± 2.43	0.209
Creatinine	50.46 ± 21.28	54.74 ± 33.29	0.503
D-Dimer	1.85 ± 2.29	1.73 ± 1.62	0.83

Data are n and mean ± SE. ^#^Student’s *t*-test.

The normal limits: White blood cell (WBC), 3.5–9.5 (×10^9^/L); Neutrophil, 1.8–6.3 (×10^9^/L); Lymphocyte, 1.1–3.2 (×10^9^/L); red blood cell count (RBC), 3.8–5.1 (×10^9^/L); Hemoglobin (HGB), 115–150 (g/L); Platelet: 125–350 (×10^9^/L); Alanine transaminase (ALT), 7–40 (U/L); Aspartate transaminase (AST), 13–35 (U/L); Albumin (ALB), 40–55 g/L; Globin (GLB), 20–40 g/L; Urea, 2.6–7.5 mmol/L; Creatinine, 41–81 (μmol/L); D-dimer: 0–1.0 (mg/L).

### Obstetric outcomes of pregnant women with COVID-19

Among 79 pregnant women with COVID-19, 14 cases were cured for COVID-19 and discharged, they delivered in other hospitals later, and their data was not available for our study. The obstetric outcomes of 65 women were collected, the median gestational age at delivery was 38 weeks, and 8 (12.3%) patients had preterm birth (Table 3) and one preterm birth was attributable to COVID-19, the other 7 cases were due to other maternal/fetal conditions. 34 (52.3%) pregnant women underwent cesarean delivery, premature rupture of membranes and preeclampsia occurred in 8 cases and 3 cases, respectively. Two patients experienced stillbirth (Table 3). There was no significant difference statistically for gestational age at delivery, the frequency of preterm birth (≤37 weeks), preeclampsia, miscarriage, stillbirth, premature rupture of membranes, and cesarean delivery between pregnant women with symptomatic COVID-19 and those with no COVID-19 symptom (Table 3).

**Table 3:** Obstetric outcomes according to COVID-19 severity.

Characteristics	Total obstetric outcome (*N* = 65)	Maternal disease severity symptomatic (*N* = 29)	Maternal disease severity asymptomatic (*N* = 36)	*P-*value
Gestational age at delivery, week	Median 38 (Range: 36–39) (IQR: 37.39)	Median 37 (Range: 30–40) (IQR: 37.39)	Median 38 (Range: 33–40) (IQR: 37.39)	0.057^$^
Preterm birth (≤37 week)	8 (12.3%)	5 (20%)	3 (8.6%)	0.450^#^
Preeclampsia	3 (4.6%)	2 (6.7%)	1 (2.9%)	0.582^#^
Miscarriage	0	0	0	
Stillbirth	2 (3.1%)	2 (6.7%)	0	0.195^#^
Premature rupture of membranes	6 (9.2%)	3 (6.7%)	3 (8.6%)	>0.999^#^
Cesarean delivery	34 (52.3%)	18 (60%)	16 (45.7%)	0.157*

*Chi-square test. ^#^Fisher exact test. ^$^Student’s *t*-test.

### Neonatal outcomes

In this study, 65 pregnant women gave birth to 68 neonates, including three sets of twins. In our study, we observed two cases of stillbirth, with confirmation of fetal demise prior to delivery. One mother experienced a fever 2 days before delivery and had comorbidities including gestational diabetes, mild thalassemia, and intrahepatic cholestasis. The other mother had preeclampsia, was carrying a set of twins, and had a fever 6 days before delivery, resulting in the death of one baby prior to birth. These two instances of stillbirth were attributed to the presence of COVID-19 infection. There were no cases of miscarriage. Of the 66 live neonates, 10 were preterm births, five of which were from three sets of twins. The Apgar scores for most neonates were 10–10 at one and 5 min after birth, except for a few cases. One mother with COVID-19 symptoms delivered two babies by cesarean section; one was stillborn, and the other suffered respiratory distress and had an Apgar score of 0–2. All other cesarean delivery neonates had Apgar scores of 10–10. Five vaginally delivered neonates had lower Apgar scores, with scores ranging from 5–10 to 8–9. Of the 66 live neonates, 10 (including three sets of twins) had low birth weights. As indicated in Table 4, the symptomatic group and asymptomatic group were compared, there was significance statistically only for the low weight neonates, there were more low weight neonates in symptomatic mother group than that of asymptomatic mother group (*p* = 0.04, Chi-square test).

**Table 4:** Neonatal outcomes according to maternal COVID-19 severity.

Characteristics	Total neonates (*N* = 66)^#^	Maternal disease severity (symptomatic) (*N* = 29)	Maternal disease severity (asymptomatic) (*N* = 37)	*P*-value
Gender				0.037
Male	36	20	16	
Female	30	9	21	
Birth weight, kg	3.06 ± 0.66	3.10 ± 0.76	3.03 ± 0.57	0.681
Normal weight (>2.5 kg)	53	20	33	0.040
Low weight (<2.5 kg)	13	9	4	
Apgar score				>0.999
1-min (≤9)	6	3	3	
5-min (≤9)	2	2	2	
Transient tachypnea of newborn	9	5	4	0.491
Respiratory distress syndrome	3	3	0	0.080
Neonatal pneumonia	6	4	2	0.392
Jaundice	34	18	16	0.129
Mechanical ventilation	1	1	0	0.439
COVID-19 positive	1	1	0	0.439
Admission at neonate unit	43	21	22	0.273

^#^Only live neonates were calculated. COVID-19, coronavirus disease 2019.

Forty three neonates were admitted to the Neonate Ward of two hospitals, with 13 admitted for COVID-19 caution and medical isolation, and 30 admitted for other medical reasons. Twenty eight neonates underwent mother-infant rooming-in and breastfeeding under the conditions of informed consent from family members and the use of personal protective measures. SARS-CoV-2 nucleic acid testing was conducted on 41 neonates daily after birth, with only one neonate testing positive for SARS-CoV-2 infection on the third day after birth. The infected neonate exhibited typical fever and acute respiratory tract syndrome but ultimately had a good prognosis, recovering after 5 days of treatment. Interestingly, neonates born of mothers with COVID-19 symptoms were more likely to have low birth weights than neonates born of mothers without COVID-19 symptoms. It is worth noting that no cases of vertical transmission of COVID-19 occurred among neonates with SARS-CoV-2 nucleic acid test results (Table 4).

## Discussion

As the pandemic spreads, the clinical characteristics of COVID-19 during pregnancy, pregnancy outcomes, and the potential risk of vertical transmission have become key concerns for obstetricians. Preliminary data suggests that the risk of placental dysfunction and fetal distress during pregnancy from Omicron infection may be lower than previous variants and Delta variant (6, 7). However, given the high transmission rate, fast transmission speed, and wide affected population, its clinical implications still warrant attention from obstetricians. No symptom associated with COVID-19 was identified in 49.4% (39/79) of our study cohort, and 40 (50.6%) cases presented with symptoms of COVID-19. Clinical manifestations of Omicron infection during pregnancy are mainly mild in our study, the most common symptoms are fever, and respiratory symptoms such as cough, nasal congestion, and sore throat are common, digestive symptoms such as nausea, vomiting, and diarrhea were rare, this result was similar to previous study (4).

The Royal College of Obstetricians and Gynecologists (RCOG) in the UK states that Omicron is milder than Delta but more infectious and may still be associated with adverse pregnancy and neonatal outcomes (8). During the Delta and Omicron variant epidemics in Scotland, a population-based cohort study was conducted on pregnant women who had been infected with SARS-CoV-2 (9). After investigating 9,923 SARS-CoV-2 infections, the study found that compared to Delta infection, pregnant women infected with Omicron during pregnancy had a lower risk of admission to the intensive care unit (0.3% vs. 1.8%), a lower rate of premature birth within 28 days after infection (1.8% vs. 4.2%), a lower rate of stillbirth within 28 days after infection, and a lower rate of neonatal infection within 28 days after birth (9). In addition, no newborn deaths were reported among pregnant women infected with Omicron while 4 newborn deaths were reported among pregnant women infected with Delta (9). In our study, 2 stillbirths occurred and 3 cases were born with respiratory distress syndrome, their mothers presented with symptom of COVID-19, these outcomes may be the consequence of COVID-19 infection.

Pregnant women infected during Omicron epidemic had a severe rate of 1.8%, compared to 13.3% during the Delta epidemic, and the proportion of those admitted to the ICU was 1.3%, significantly lower than the 17.7% during the Delta epidemic (10). The clinical presentation of our study cohort was generally mild, and there was no pregnant woman infected with omicron variant admitted to the ICU. However, due to physiological changes during pregnancy such as significantly increased blood volume, decreased albumin concentration (as identified in our study), and increased susceptibility to hormone-induced liver toxicity, pregnant women may experience changes in immune suppression, making them more susceptible to severe illness if they become infected with SARS-CoV-2, this increases the risk for both the mother and the fetus (10).

COVID-19 is an illness that affects multiple organs in addition to the lungs and elicits an increased inflammatory response in the host. The liver is one such organ that can be affected by this systemic disease. Specifically, our study found that 11.4% (9/79) of COVID-19 infected women had liver biochemistry abnormalities upon admission, and 20% (8/40) of symptomatic women presented abnormal results, which were reflected through elevated levels of AST and ALT. The elevation in AST and ALT levels have previously been linked with an increased risk of mortality and complications, serving as an indicator of the severity of COVID-19 (11). In our study, our observations showed that one of the mothers experienced stillbirth and displayed the highest AST and ALT levels among the study cohort. Another baby she delivered had respiratory distress, requiring intubation after birth. This finding suggests that the hepatic involvement of pregnant women with COVID-19 may imply an increased risk of adverse fetal complications, including respiratory distress and stillbirth.

Several systematic reviews and cohort studies in different regions have included pregnant women infected with SARS-CoV-2 in early pregnancy and tracked their pregnancy outcomes, finding no increase in the natural miscarriage rate with early infection. A prospective cohort study in the USA (12) included 94 early pregnant women who tested positive for COVID-19 before 14 weeks and had symptoms. Follow-up showed that early pregnancy infection with COVID-19 did not increase the rate of natural miscarriage before 20 weeks. An Italian case–control study (13) included 225 pregnant women, of whom 23 had COVID-19 symptoms in early pregnancy. Multivariate analysis showed that early pregnancy COVID-19 infection was not an independent risk factor for natural miscarriage (13). Our study only included 5 cases of early pregnancy, there was no miscarriage occurrence.

Many studies and systematic reviews suggest that pregnant women with SARS-CoV-2 infection have increased rates of premature birth. Maternal fever and hypoxemia can increase the risk of premature labor and premature rupture of membranes, leading to an increase in spontaneous and medically-induced premature birth (14, 15). A large multicenter cohort study in the US showed that early and late pregnancy SARS-CoV-2 infection resulted in premature birth rates of 11.8 and 17.8%, respectively, compared to an overall premature birth rate of 10.0–10.2% in the US from 2018 to 2020 (16). Premature birth rate of our study cohort was 12.3%, similar to the data from US.

Population-based cohort studies have reported an increased risk of severe complications during pregnancy in patients infected with SARS-CoV-2. The risks of hypertensive disorders in pregnancy and postpartum hemorrhage increased, and maternal deaths occurred in the COVID-19 group (17). A meta-analysis showed that the risks of preeclampsia, premature birth, and stillbirth increased in women infected with SARS-CoV-2 during pregnancy (18, 19). Only two stillbirths occurred, there was no severe case and maternal death in our study cohort.

It is important to note that advanced maternal age itself is associated with various underlying factors that can contribute to adverse outcomes. These factors may include pre-existing medical conditions, increased comorbidity, decreased immune function, and decreased physiological reserves. When combined with COVID-19 infection, these factors may further exacerbate the risk of adverse outcomes in pregnant women. Advanced maternal age has been identified as a potential risk factor for adverse outcomes in pregnancies complicated by COVID-19 infection (20). Several studies have reported an increased risk of adverse outcomes, such as preterm birth, preeclampsia, and cesarean delivery, in pregnant women of advanced maternal age who contract COVID-19 (21, 22). Our study showed that symptomatic women had higher mean age compared to asymptomatic women, this study contribute to the body of evidence suggesting a correlation between advanced maternal age and increased risk of symptomatic outcomes in COVID-19-infected pregnancies.

There is emerging evidence suggesting that vaccination provides a protective effect against symptomatic COVID-19 infection, including the Omicron variant, in pregnant individuals (18). Several studies have indicated that COVID-19 vaccination can reduce the risk of severe illness, hospitalization, and adverse outcomes in pregnant women (9, 14, 18). These findings support the notion that vaccination can offer protection in pregnant individuals. However, there was no difference statistically between the symptomatic group and the asymptomatic group for vaccination status in our study. Based on our limited sample size, it appears that vaccination may not have an effect in preventing symptomatic infection of COVID-19 Omicron. This could potentially be attributed to the long duration (at least 8 months) between vaccination and infection, as the protective effect may diminish over time.

The data from this cohort of women infected with the Omicron variant indicate a relatively high cesarean section rate (52.3%). This finding is consistent with previous reports (23). It is important to consider this observation in the context of the milder presentation of the Omicron variant compared to earlier variants. The milder presentation of the Omicron variant may influence the decision-making process regarding the mode of delivery. In some cases, healthcare providers may opt for a cesarean section as a precautionary measure, considering the potential risks associated with vaginal delivery in the context of COVID-19 infection. Additionally, factors such as maternal symptoms, disease severity, and obstetric indications may also contribute to the decision-making process. It is worth noting that cesarean section has been reported to have an impact on breastfeeding rates in women (24). This is primarily due to the potential challenges in initiating and establishing breastfeeding after a surgical delivery. However, it is important to emphasize that each case should be carefully evaluated, keeping in mind the specific circumstances and individualized care of the mother and infant.

As the COVID-19 pandemic in China continues, new challenges have emerged in its prevention and control. These include the mutation of the SARS-CoV-2 virus, changes in the epidemic situation, the need to promote vaccination, and the accumulation of experience in prevention and control measures. The management of COVID-19 during pregnancy is also a pressing matter that requires the summarization of experiences and the development of new management strategies. To address these issues, there is a need for more high-quality, multidisciplinary, and multicenter studies to investigate various aspects related to SARS-CoV-2 infections in pregnant women, such as their impact on maternal and fetal outcomes, the safety and effectiveness of therapeutic drugs, and the long-term growth and development of newborns. Conducting such research will be crucial to enhance clinical research on pregnant women with COVID-19 in China.

Indeed, it is essential to consider a wide range of data when studying COVID-19, particularly including maternal-fetal outcomes. However, it’s worth noting that the current study has several limitations. Firstly, the sample size of patients analyzed in the manuscript is relatively small, which may limit the generalizability of the findings. A larger sample size would provide more robust and representative results. Secondly, the study does not include a comparison between different variants of COVID-19, such as delta, beta, and omicron, within the same study population. The omission of such a comparison restricts our understanding of the potential impact of these variants on maternal-fetal outcomes. Comparing outcomes across different variants would provide valuable insights into their respective risks and effects on pregnancy (25). Additionally, the study may not account for potential confounding variables that can influence maternal-fetal outcomes. Factors like socioeconomic status, and access to healthcare could significantly impact the results. The absence of controlling for these variables limits the ability to attribute observed outcomes solely to the impact of COVID-19. Lastly, it is important to consider the potential biases in patient selection, as the study may have included patients from a specific region or hospital, possibly leading to a non-representative sample that could affect the generalizability of the findings to other populations. Addressing these limitations and conducting larger, well-controlled studies that include comparisons across different COVID-19 variants would help improve our understanding of maternal-fetal outcomes during the pandemic.

In summary, our study, which is based on a limited sample size, suggests that the risk of placental dysfunction and fetal distress during pregnancy from Omicron infection may be lower than with previous variants. However, it also indicates that adverse pregnancy outcomes, including stillbirth, premature birth, fetal distress, and cesarean section rate, may be affected by SARS-CoV-2 Omicron infection.

## Data availability statement

The original contributions presented in the study are included in the article/supplementary material, further inquiries can be directed to the corresponding authors.

## Ethics statement

The studies involving humans were approved by the Ethics Committees of People’s Hospital of Yangjiang and Chaozhou Central Hospital. The studies were conducted in accordance with the local legislation and institutional requirements. The ethics committee/institutional review board waived the requirement of written informed consent for participation from the participants or the participants’ legal guardians/next of kin because the patient’s data were analyzed anonymously and this was a retrospective study.

## Author contributions

L-YY conceived, designed the study, and wrote the manuscript. L-HL, Y-KY, C-FL, and R-ZZ performed the clinical practices and collected the data. L-YY, L-LL, and FL analyzed the data and wrote the manuscript. L-LL, LZ, Y-WL, G-KZ, Y-QZ, Y-CH, and Y-BC collected the data. All authors read and approved the final manuscript.

## Funding

This study was supported by the Special Research Plan 2019 of Chaozhou (grant no. 2020xg01), High Level Development Plan of People’s Hospital of Yangjiang (grant no. G2020007), and Research Plan of Hygiene and Health Bureau of Yangjiang (grant no. 2023032). The funder had no role in the study’s design, data interpretation, and manuscript writing.

## Conflict of interest

The authors declare that the research was conducted in the absence of any commercial or financial relationships that could be construed as a potential conflict of interest.

## Publisher’s note

All claims expressed in this article are solely those of the authors and do not necessarily represent those of their affiliated organizations, or those of the publisher, the editors and the reviewers. Any product that may be evaluated in this article, or claim that may be made by its manufacturer, is not guaranteed or endorsed by the publisher.
